# Metabolomics as a Tool for Discovery of Biomarkers of Autism Spectrum Disorder in the Blood Plasma of Children

**DOI:** 10.1371/journal.pone.0112445

**Published:** 2014-11-07

**Authors:** Paul R. West, David G. Amaral, Preeti Bais, Alan M. Smith, Laura A. Egnash, Mark E. Ross, Jessica A. Palmer, Burr R. Fontaine, Kevin R. Conard, Blythe A. Corbett, Gabriela G. Cezar, Elizabeth L. R. Donley, Robert E. Burrier

**Affiliations:** 1 Stemina Biomarker Discovery, Madison, Wisconsin, United States of America; 2 The M.I.N.D. Institute and Department of Psychiatry and Behavioral Sciences, University of California Davis, Davis, California, United States of America; 3 The Jackson Laboratory for Genomic medicine, University of Connecticut Health Center, Farmington, Connecticut, United States of America; 4 Department of Psychiatry, Psychology and Kennedy Center, Vanderbilt University, Nashville, Tennessee, United States of America; Biogen Idec, United States of America

## Abstract

**Background:**

The diagnosis of autism spectrum disorder (ASD) at the earliest age possible is important for initiating optimally effective intervention. In the United States the average age of diagnosis is 4 years. Identifying metabolic biomarker signatures of ASD from blood samples offers an opportunity for development of diagnostic tests for detection of ASD at an early age.

**Objectives:**

To discover metabolic features present in plasma samples that can discriminate children with ASD from typically developing (TD) children. The ultimate goal is to identify and develop blood-based ASD biomarkers that can be validated in larger clinical trials and deployed to guide individualized therapy and treatment.

**Methods:**

Blood plasma was obtained from children aged 4 to 6, 52 with ASD and 30 age-matched TD children. Samples were analyzed using 5 mass spectrometry-based methods designed to orthogonally measure a broad range of metabolites. Univariate, multivariate and machine learning methods were used to develop models to rank the importance of features that could distinguish ASD from TD.

**Results:**

A set of 179 statistically significant features resulting from univariate analysis were used for multivariate modeling. Subsets of these features properly classified the ASD and TD samples in the 61-sample training set with average accuracies of 84% and 86%, and with a maximum accuracy of 81% in an independent 21-sample validation set.

**Conclusions:**

This analysis of blood plasma metabolites resulted in the discovery of biomarkers that may be valuable in the diagnosis of young children with ASD. The results will form the basis for additional discovery and validation research for 1) determining biomarkers to develop diagnostic tests to detect ASD earlier and improve patient outcomes, 2) gaining new insight into the biochemical mechanisms of various subtypes of ASD 3) identifying biomolecular targets for new modes of therapy, and 4) providing the basis for individualized treatment recommendations.

## Introduction

Autism spectrum disorder (ASD) is a lifelong neurodevelopmental disorder characterized by social deficits, impaired verbal and nonverbal communication and repetitive movements or circumscribed interests [1]. About 1 in 68 children has been identified with autism spectrum disorder (ASD) according to estimates from CDC's Autism and Developmental Disabilities Monitoring (ADDM) Network. The current process for a clinical diagnosis includes establishing a developmental history and assessments of speech, language, intellectual abilities, and educational or vocational attainment. In practice, these methods lead to a diagnosis at an average age of 4 years [2] in the United States. It is recognized that establishing personalized therapy for children with ASD at the earliest age possible improves outcomes including a higher level of cognitive and social function and improved communication as well as decreased financial and emotional burden on families [3], [4]. Development of blood-based diagnostic tests to aid in the assessment of risk for a diagnosis of ASD at an early age would facilitate implementing intensive behavioral therapy at the earliest age possible.

The etiology of the vast majority of cases of ASD are unknown and their genetics have proven to be incredibly complex [5], [6]. There is now widespread appreciation that there will be many causes of ASD with varying combinations of genetic and environmental risk factors at play. Numerous studies have attempted to identify the causes of the disorder by studying transcriptomics and genomics, leading to the identification of multiple genes associated with ASD [6], [7]. There are currently hundreds of observable genetic variants that account for about 20% of the cases of autism. These data are currently most useful in understanding the intra-familial genetics of autism. For this reason, clinical tests based on genomic measures often include genetic counseling to assess the chance of disease occurrence or recurrence within a family [8], [9]. Prediction accuracies of ASD risk based on genomic approaches range from 56% to 70% depending largely on the population of patients assessed. Separate analyses of at least one of the genomic studies by Skafidas *et al*. has questioned whether the results have been confounded by biases due to ancestral origins [10], [11]. An additional limitation of genomic studies is that the results of environmental influences on the child and/or mother are not discernible. Metabolomics is sensitive to biochemical changes caused by even subtle environmental influences and therefore can complement genomic approaches by addressing some of these factors that may be closer to phenotype.

Given the complexities of the genetic environment of ASD, metabolomic profiling may provide an alternative path to developing early diagnostic tests. Previous metabolic studies of ASD have used biological matrices such as cells, organelles, urine and blood, and have implicated a wide range of metabolites including fatty acids, sterols, intermediary metabolites, phospholipids, and molecules associated with oxidative stress [12]–[16]. Two recent reports highlight the potential use of metabolomic analysis of urine to identify signatures of ASD. One study used 1H-NMR methods and showed changes in metabolites associated with the tryptophan/nicotinic acid metabolic pathway, sulphur and amino acid pathways, as well as microbial metabolites implicating the involvement of microbial metabolism in the etiology of ASD [16]. Ming *et al*. used a combination of liquid- and gas-chromatography based mass spectrometry methods to identify changes in a number of amino acids and antioxidants such as carnosine, as well as confirming the changes associated with altered gut microbiomes [17].

Measurement of metabolites offers an excellent opportunity to identify differences in small molecule abundance that may have the ability to characterize some forms of ASD. High resolution mass spectrometry (HRMS) is not only a very sensitive detection method for small molecule metabolites, it also provides accurate mass data that aids in metabolite identification through molecular formulae determination [18]. HRMS offers an additional distinct advantage in the ability to distinguish between compounds with the same nominal mass (isobaric compounds), providing enhanced chemical formula and structure information [19]. Unfortunately there is not one universal chromatographic mass spectrometric technique capable of detecting all of the metabolites in blood. To identify novel potential biomarkers associated with ASD, it is necessary to facilitate broad metabolite detection coverage. Toward this goal, we applied an orthogonal approach to chromatographic separation, mass spectral ionization and detection [20]. The current study employed multiple chromatographic mass spectrometric metabolomic methods including gas chromatography-mass spectrometry (GC-MS) and liquid chromatography-high resolution mass spectrometry (LC-HRMS) to discover a wide range of metabolites in blood plasma samples that were able to differentiate TD individuals from those with ASD. Subsequently, tandem mass spectrometry (MS-MS) experiments were employed to aid in structural confirmation of the metabolites discovered by LC-HRMS.

The aim of the study was to perform a broad evaluation of small molecules in blood plasma to discover metabolites that may lead to biomarkers associated with ASD. Univariate, multivariate and machine learning methods were employed to discover metabolites or groups of metabolites exhibiting statistically significant abundance differences that can be used as biomarkers to distinguish children with ASD from TD individuals.

## Materials and Methods

### Subject Samples

The experimental subjects were initially recruited through the UC Davis M.I.N.D. Institute Clinic, Regional Centers, referrals from clinicians, area school districts and community support groups such as Families for Early Autism Treatment (FEAT). Subjects were limited to an age range of 4–6 years. Typically developing participants were recruited from area school districts and community centers. All facets of this study were approved by the University of California at Davis Institutional Review Board (IRB). Written informed consent was obtained from the parent or guardian of each participant and data were analyzed without personal information identifiers then subjects completed diagnostic and psychological measures. Study participants with ASD were enrolled under inclusion criteria consisting of a diagnosis of autism spectrum disorder based on the DSM-IV criteria determined by an experienced neuropsychologist (BAC), which was further corroborated by the following measures using research reliable clinicians: the Autism Diagnostic Observation Schedule-Generic (ADOS-G) provides observation of a child's communication, reciprocal social interaction, and stereotyped behavior including an algorithm with cutoffs for autism and autism spectrum disorder; the Autism Diagnostic Interview-Revised (ADI-R) is a comprehensive, semi-structured parent interview that assesses a child's developmental history and relevant ASD characteristic behaviors and generates a diagnostic algorithm for children with ASD. Based on the DSM-IV criteria [21], only children with strictly defined autistic disorder were enrolled whereas children with pervasive developmental disorder-not otherwise specified (PDD-NOS) or Asperger Syndrome were excluded from the study. The Social Communication Questionnaire (SCQ) was used as a screening tool to ensure the absence of symptoms of ASD in the TD children. The patients recruited for this study were primarily Caucasian and the ages were similar between groups. However, the participants with autism had lower IQ scores than the TD subjects [22], [23].

The exclusion criteria for all subjects included the presence of Fragile X or other serious neurological (e.g., seizures), psychiatric (e.g., bipolar disorder) or known medical conditions such as autoimmune disease and inflammatory bowel diseases/celiac disease. All subjects were screened via parental interview for current and past physical illness. Children with known endocrine, cardiovascular, pulmonary, and liver or kidney disease were excluded from enrollment in the study. Dietary restriction for participation in the study was not required with the exception of an overnight fast. Participation in the study required two clinical visits for behavioral assessment and blood draws. After application of exclusion criteria, the final study group consisted of 104 children, 69 with ASD and 35 in the TD group.

Samples were collected on Thursday morning visits to the M.I.N.D. Institute over a period of 13 months. Blood was drawn into a 9.6 mL EDTA Vaccutainer tube by an experienced pediatric phlebotomist between the hours of 8 and 10 AM following an overnight fast. Tubes were immediately inverted 6–8 times to assure mixing with the anticoagulant and placed on ice. Immediately after plasma separation and aliquoting, samples were sent on the morning of the draw via courier with a barcode label, wrapped tube cap with a strip of parafilm; bubble wrapped then set in a biohazard bag which was placed inside a carrier between coolant packs. Samples were stored at −80°C.

Samples from 103 of the 104 children were sent to Stemina for metabolomic analyses on dry ice… Upon receipt, 5 samples were removed after visual inspection and observation of overt hemolysis and the remaining 98 samples were analyzed by mass spectrometry. Quality checks of the raw mass spectrometric data from the 98 samples were performed, resulting in removing data from 16 patient samples that did not contain MS data from all 5 methods from further analysis. The final 82 samples used in these studies originated from 52 children with ASD and 30 children in the TD group. The children were chosen so that the age and gender distributions were similar across the groups. There was no statistical difference in age between ASD cases and the TD children for the current study (Welch's t-test p = 0.25) (see Table 1).

**Table 1:** Patient demographic information.

Demographic		TD	ASD	Overall
Group Size		30	52	82
Sex (male %)		86.67	78.85	81.7
	Range	4.17–6.92	4–6.92	4–6.92
Age (Years)	Average	5.6	5.37	5.46
	Std. Dev.	0.95	0.81	0.87
	Range	88–137	40–110	40–137
IQ	Average	114.3	67.48	80
	Std. Dev.	10.78	17.69	27.47

Regarding patient medication, 18 out of 52 of the subjects with ASD in this study were taking medications which included Risperidone (5), Sertraline (3), Aripiprazole (2), antihistamines (2), antivirals (2), antifungals (2), and various other less frequent drugs. Three of the 30 typical subjects were taking medications, which included methylphenidate (1), albuterol (1) and loratadine (1). Ten of the 52 ASD subjects were on a gluten and/or casein-free (GFCF) diet. Importantly, blood draws were administered prior to eating and any morning administration of any medication.

### Sample Preparation for LC-MS

Plasma samples were split into 50 µl aliquots and stored at −80°C prior to metabolite extraction. Samples were kept on ice during these procedures. Samples were randomized into three batches for the LC-HRMS analysis such that diagnosis, IQ, age and ethnicity were equally distributed in each batch. Small molecules were extracted from 50 µL plasma aliquots using 450 µL of 8∶1 methanol: water solution at −20°C [24]. The extraction solution also contained internal standards. The samples were agitated for 10 minutes at 2–8°C then centrifuged at 18,400×G for 20 minutes at 4°C to remove the precipitate. The supernatant was transferred to a fresh tube and the centrifugation step was repeated to remove any residual precipitate. After the final centrifugation, 450 µL of supernatant was transferred to a fresh tube then evaporated to dryness in a SpeedVac, then resolublized in 45 µL of a 50∶50 mixture of 0.1% formic acid in acetonitrile: 0.1% formic acid, also containing internal standards. This solution was then transferred to a high performance liquid chromatograph (HPLC) autosampler injection vial for LC-HRMS analysis.

### Mass Spectrometry

Both targeted GC-MS as well as untargeted LC-HRMS were employed for better metabolome coverage. Four untargeted LC-HRMS methods were used including C8 or HILIC chromatography coupled to electrospray ionization in both positive and negative ion polarities, resulting in 4 separate data acquisitions per sample. For each methodology and condition, only a single sample aliquot was assessed, due to limited material availability.. LC-HRMS methods were developed and tested prior to the evaluation of the clinical patient samples to optimize the breadth of coverage of small molecule metabolites.

### Liquid Chromatography High Resolution Mass Spectrometry

LC-HRMS was performed using an Agilent G6540 Quadrupole Time of Flight (QTOF) system consisting of an Agilent 1290 HPLC coupled to a high resolution (QTOF) mass spectrometer. Electrospray ionization (ESI) in both positive and negative ion modes was employed using a dual ESI source under high-resolution exact mass conditions. 2 µL of sample was injected. A Waters Acquity ultra high performance liquid chromatography (UPLC) BEH Amide column with dimensions 2.1×150 mm, 1.7 µM particle size was used for Hydrophilic Interaction Liquid Chromatography (HILIC), and maintained at 40°C. Data was acquired for each sample for 29 minutes at a flow rate of 0.5 mL/minute using a solvent gradient with 0.1% formic acid in water and 0.1% formic acid in acetonitrile. An Agilent Zorbax Eclipse Plus C8 2.1×100 mm, 1.8 µM particle size column was used for C8 chromatography and data was acquired for each sample for 50 minutes at a flow rate of 0.5 mL/minute using a gradient with 0.1% formic acid in water and 0.1% formic acid in acetonitrile and maintained at 40°C.

### Gas Chromatography - Mass Spectrometry

GC-MS analyses were performed at the West Coast Metabolomics Center at UC Davis as described in [25]. GC-MS data was acquired using an Agilent 6890 gas chromatograph coupled to a LECO Pegasus IV TOF mass spectrometer. Metabolite identification was done by comparing sample data to a database of over 1,000 compounds identified by GC-MS that includes mass spectra, retention indices, structures and links to external metabolic databases.

### Metabolite chemical structure confirmation by LC-HRMS-MS

The chemical structures of key metabolites were further confirmed using tandem mass spectrometry (LC-HRMS-MS) methods with chromatographic conditions identical to those used for their discovery. LC-HRMS-MS analyses were performed on an Agilent QTOF mass spectrometer for patient samples and/or, reference blood samples with collision energy conditions optimized to obtain the highest quality product ion spectra. The resulting product ion spectra were then compared to MS-MS spectra available in public spectral databases such as METLIN [26], MassBank [27] and Stemina's own SteminaMetDB database.

### Data Analysis

#### LC-HRMS Data preprocessing

Raw mass spectral data and were initially examined for quality criteria established during method development such as abundance thresholds, retention time and peak shape consistency for total ion chromatograms, and extracted ion chromatograms for internal standards and markers. Data files exhibiting chromatograms that failed these quality criteria were removed from further analysis. A portion of these were retested, depending on the nature of the QC failure. Raw data were converted to open source mzData files [28]. Peak picking and feature creation were performed using XCMS [29] and then deviations in retention times were corrected using the obiwarp algorithm [30] based on a non-linear clustering approach to align the LC-HRMS data. Mass features were generated using the XCMS density based grouping algorithm. Missing features were integrated based on retention time and mass range of a feature bin using iterative peak filling. A “mass feature” (also abbreviated here as “feature”) is a moiety detected by the mass spectrometer that is defined by 2 properties 1) the detected mass-to-charge ratio (*m/z*) and 2) the chromatographic retention time.

A series of data filters were then employed to remove features exhibiting low abundance levels, those resulting from background noise, ions with non-biological mass defects, and known contaminants from subsequent data analyses. To reduce LC-HRMS batch variations in feature detection, the abundance values were then normalized by sample to the experiment-wide median area of spiked-in internal reference standards. The integrated areas of the normalized mass features from the GC-MS and LC-HRMS platforms were combined into a single dataset. There were 4572 features for the training set of samples that passed preprocessing filters.

#### Training and Independent Validation Sets

The 82 patient samples (52 ASD and 30 TD samples) were split into two sets, (1) a training set of 61 samples (39 ASD and 22 TD) for identification of statistically significant features and classification modeling and (2) a 21-sample independent validation set (13 ASD and 8 TD) used to evaluate performance of the classification models. This was accomplished by randomizing the samples using the diagnosis, patient IQ, and gender in these training and validations sets so that each set contained a similar proportion of factors used in randomization. The validation sample set was withheld from the univariate filtering and model development process to act as an independent external sample set to evaluate model performance. Detailed patient demographics for the samples in the training and validation sets are provided in Table S4.

#### Univariate Filtering of Mass Features

T-tests were used to reduce the overall feature set, the potential for over-fitting, and increase the biological interpretability of the predictive signature [31]. The integrated areas of mass features normalized to internal standards (IS) from the GC-MS and LC-HRMS platforms were combined into a single dataset. The 4572 features passing the preprocessing filters for the training set of samples were further filtered using Welch T-tests under the null hypothesis that no difference in mean integrated areas of a mass feature is present between the experimental classes, and the alternative hypothesis that there is a difference in mean integrated areas between ASD and TD training set samples to identify differential features. For each feature that exhibited a statistically significant change with an uncorrected p-value <0.05, its extracted ion chromatogram (EIC) was reviewed for consistency of integration across samples, peak shape, and a minimum peak height requirement of >3000. Features passing this EIC quality review process were then utilized in the classification modeling. False discovery rates (FDRs) were calculated using the Benjamini Hochberg method of p-value correction [32].

#### Classification Modeling

Model development was performed with two primary goals: 1) to robustly rank the importance of metabolites in discriminating ASD using a VIP (Variable Importance in the Projection) score index and 2) to identify the minimum set of predictive metabolites needed to reach the highest levels of differentiation of the ASD and TD experimental classes. The final models were created by training a Partial Least Squares Discriminant Analysis (PLS-DA) or Support Vector Machine (SVM) classifier using the entire 61-sample training set. The modeling techniques PLS-DA as well as SVM with a linear kernel [33], [34] were both utilized to demonstrate that the molecular signature can be predictive using multiple approaches. PLS and SVM classification models were created using the R package Classification and Regression Training “caret” version 5.17–7 [35]. Receiver operator Curve (ROC) analysis was performed using the R package ROCR version 1.0–5 [36].

**Figure 1:**
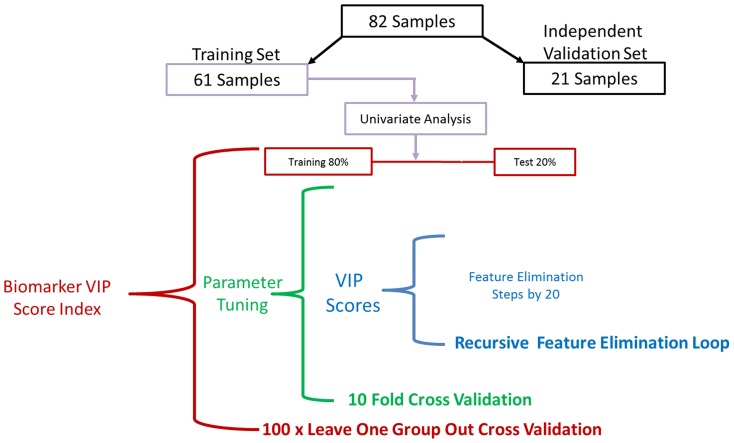
Classification modeling process. A three-layer nested cross-validation approach was applied using both PLS-DA and SVM modeling methods to determine significant features capable of classifying children with ASD from TD children. The 179 features of the training set were analyzed using a leave-one-group-out cross-validation loop as described. The results from this cross-validation process were used to estimate model performance and create a robust feature VIP score index to rank the ASD vs TD classification importance of each of the 179 features. These feature ranks were used to evaluate the performance of the molecular signature using an independent validation set.

A nested cross validation (CV) approach (Figure 1) was used to meet the first objective of model development - a robust measure of feature VIP scores. The 179 features from the 61 sample training set were analyzed using 100 resamples with an 80∶20 split to weight the importance of each of the 179 statistically significant features. The tuning loop utilized 10-fold cross validation to tune model parameters (cost parameter C for SVM and the number of components for PLS-DA). The recursive feature elimination loop was used to identify the best performing feature subset from each iteration using steps of 20 features. The results from the 100 resamples were used to estimate model performance and create a robust biomarker VIP score index to rank the importance of each of the 179 features in classification of ASD from TD individuals.

Feature VIP robustness was measured by resampling the training set 100 times using an 80∶20 split into 49-sample CV training and 12-sample CV test sets. VIP scores were calculated for each of the 100 resamples and the most informative features at each resample were identified by backwards recursive feature elimination (in 20-feature steps) using the Area Under the ROC Curve (AUC). The most informative set of features was then used to predict each CV test set. The VIP scores were averaged across the 100 resamples to create the VIP index for each feature. The classification performance metrics of the CV test sets were averaged across resamples to understand potential future performance.

The second objective of the classification modeling approach was to identify the minimum number of features with the highest level of classification accuracy. This objective was met using feature subsets based on the ordered VIP score index and evaluating the subset performance in the validation set of samples. The classification models were created using the entire 61 sample training set and by stepping through features. The feature stepping process utilized the 20 top VIP features then added the next 20 highest weighted features until all 179 features were evaluated.

**Figure 2:**
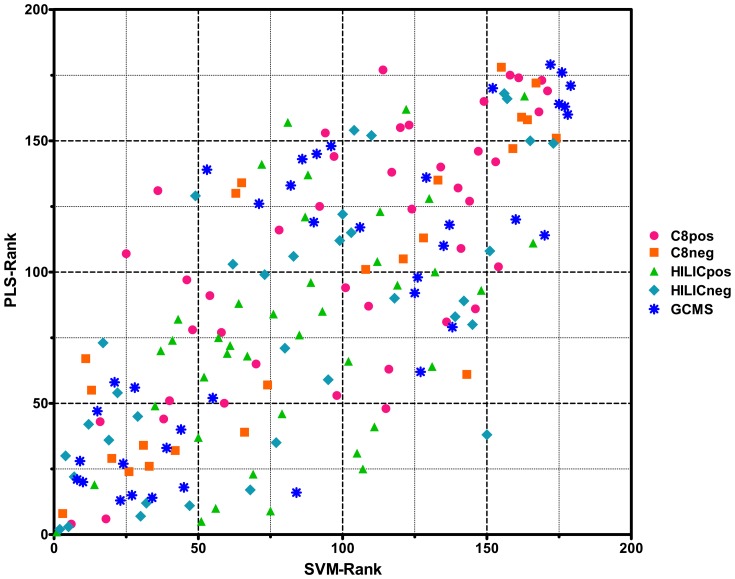
Feature Importance Rankings. The top 179 features were compared for rank between SVM and PLS modeling methods. The lowest rank scores represent the most important features.

Performance metrics (Accuracy, Sensitivity, Specificity, and ROC analysis) were determined based on the prediction of the 21 sample independent validation set for assessment of the molecular signature at each feature subset bin size (Table S3). Accuracy is defined as the proportion of correctly classified participants and is calculated by dividing the number of correctly classified participants by the total number of participants in a sample set. Specificity is the proportion of correctly classified TD individuals out of all TD participants in a sample set. Sensitivity is the proportion of correctly classified ASD individuals out of all participants with ASD in a sample set. The top 179 features were also compared for rank between SVM and PLS modeling methods (Figure 2).

#### Feature Metabolite Annotations

Metabolite annotation (assignment of putative chemical structures) was carried out for each feature. Annotation was accomplished by comparing *m/z* value of each mass feature to the *m/z* value of common ESI adducts contained in public chemical databases and/or Stemina's internal metabolite database. All mass features that were annotated with chemical identities in that the measured exact mass was consistent (within 20 ppm relative mass error) with one or more chemical structures. These annotations were considered to be putative until the chemical structure of the feature was further confirmed by LC-HRMS-MS.

The molecular formulae of the mass features with putative annotations were then input into the “Find by Formula” (FBF) algorithm in the Agilent Technologies MassHunter Qualitative Analysis software which tests whether the mass spectra for a given feature is a reasonable match with the proposed formula. In most cases, the annotations for any feature with a median FBF score of less than 70, a retention time difference greater than 35 seconds or which was present in less than 50% of the data files was not included for further analysis due to lack of confidence in the formula assignment of the annotation.

Features from the GC-MS analysis were identified as described by [25]. This procedure used comparison of the sample data to spectra of metabolite reference standards that had been previously acquired by the same identical GC-MS method. Therefore, the data analysis and confirmation of the metabolite chemical structures was performed by a simple comparison of the acquired patient sample data to the database. GC-MS data also contained peaks that remained unidentified and showed statistically significant changes depending on sample class.

## Results

The use of multiple orthogonal analytical methods provided a broad coverage of the metabolome and each method contributed mass features to the model for classification of the children with ASD from the TD controls. Each analytical method was assessed for the unique features it provided. The HILIC LC-HRMS method resulted in the highest number of distinctive mass features in the models, followed by C8 LC-HRMS then GC-MS. Univariate analysis filtering was performed on the 4572 features that passed the preprocessing filters. About 60% of the LC-HRMS features were putatively annotated with a chemical structure and 8% (503) of the annotated features passed the FBF procedural criteria. Approximately 36% (142) of the targeted GC-MS features were confirmed metabolites. A breakdown of these results is contained in Table 2.

**Table 2:** A breakdown of the numbers of features resulting from filtering and annotation processes, based on molecular formula.

Platform	Raw Features	Annotated Features	Unique Formula within a Platform	Features Passing Preprocessing Filters	Features Passing Univariate Filter
HILIC +	3207	1985	146	1527	40
HILIC−	1865	1061	140	950	35
C8+	3062	1902	140	1096	42
C8−	1568	847	77	514	23
GC-MS	485	178*	142*	485	39
**Total**	**10187**	**5795**	**645**	**4572**	**179**

This table also helps to illustrate the orthogonality and contribution of each of the 5 analytical platforms. Molecular formulae are being used here only to approximate the method orthogonality, since any given molecular formula may be associated with multiple chemical structures. *These annotations were confirmed in the GCMS platform and the formula were confirmed by using the KEGG database instead of the FBF procedure used in the 4 LCMS platforms.

Data across the 61-sample training set from all analytical platforms were used to identify and robustly rank the features that could be utilized to discriminate plasma samples from children with ASD from samples from TD children. The univariate analysis filtering, as described in the methods, resulted in 389 statistically significant features. Following feature QC, 210 features were removed from the analysis due to poor quality EICs, leaving 179 features that were included in classification modeling. The 179 features comprised 3% of the LC-HRMS and 8% of the GC-MS preprocessed set of features and are shown in Table S1.

### Training Set Model Performance

**Figure 3:**
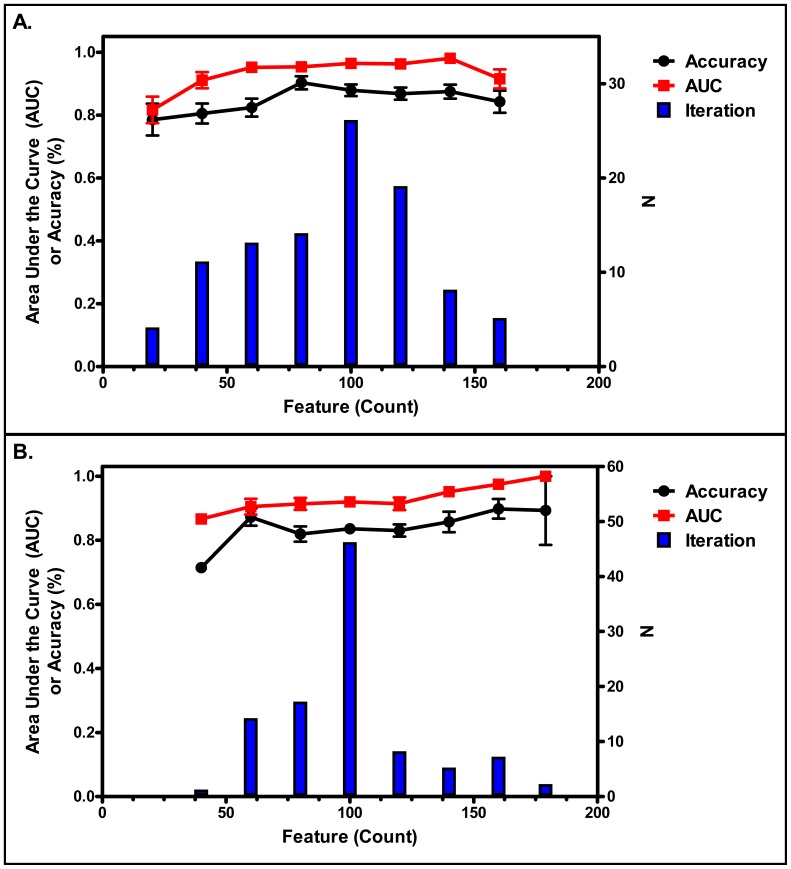
Performance of the SVM and PLS models. Average AUC and accuracy of the (a) SVM and (b) PLS models containing different numbers of features. The bar graphs show the number of optimal models which were derived from recursive feature elimination process that was included in the resampling process for the indicated number of features.

SVM and PLS classification methods were used to discriminate between samples from children with ASD and TD children using the 179 selected features as variables and each feature's contribution toward classification was evaluated for future biomarker development efforts. Based on the best performing model from each of the 100 nested CV resampling iterations, ROC plots were generated for the average of the 100 resamples to understand performance of each modeling method (SVM and PLS-DA). Both SVM and PLS modeling methods indicated that a metabolic signature could be detected that could classify children with ASD from TD individuals (Table S2). For the 61-sample training set, the average ASD prediction accuracy of the SVM model was 0.86, with AUC values of 0.95 (95% CI 0.94–0.96). The PLS model gave an average prediction accuracy of 0.84 with AUC values of 0.92 (95% CI 0.91–0.94). To confirm that the model classification accuracies were not random results, the modeling process was repeated with random permutations of the diagnosis class labels. These results showed near random classification, with AUC values of 0.52 (95% CI 0.48–0.57) and 0.52 (95% CI 0.49–0.56) for SVM and PLS, respectively, indicating that the 179 features did not discriminate the classes by chance (Figure 3).

Anticipating that blood tests for ASD may be more efficient and less expensive if they measure an optimally lower number of metabolites, the classification modeling paradigm also included a feature number optimization in each model, based on the highest resulting AUC. The feature sets were evaluated using feature subsets based on the ordered VIP scores of individual features to identify the minimum number of features that maximized performance for each modeling method (Table S2). These data together indicate that not all of the features contributed equally to the models and that the number of features could be reduced by removing those that contributed less while still retaining model accuracy and robustness. As a result, the entire set of 179 features was not required for optimal model performance for either of the modeling methods (Figure 3). The results from the model training process indicated that SVM models that were trained using an 80 feature set exhibited the best combined classification performance metrics (when compared to PLS and other SVM results) with an average accuracy of 90%, an average sensitivity of 92%, an average specificity of 87%, and an average AUC of 0.95 (Table S2).

### Validation Set Model Performance

**Figure 4:**
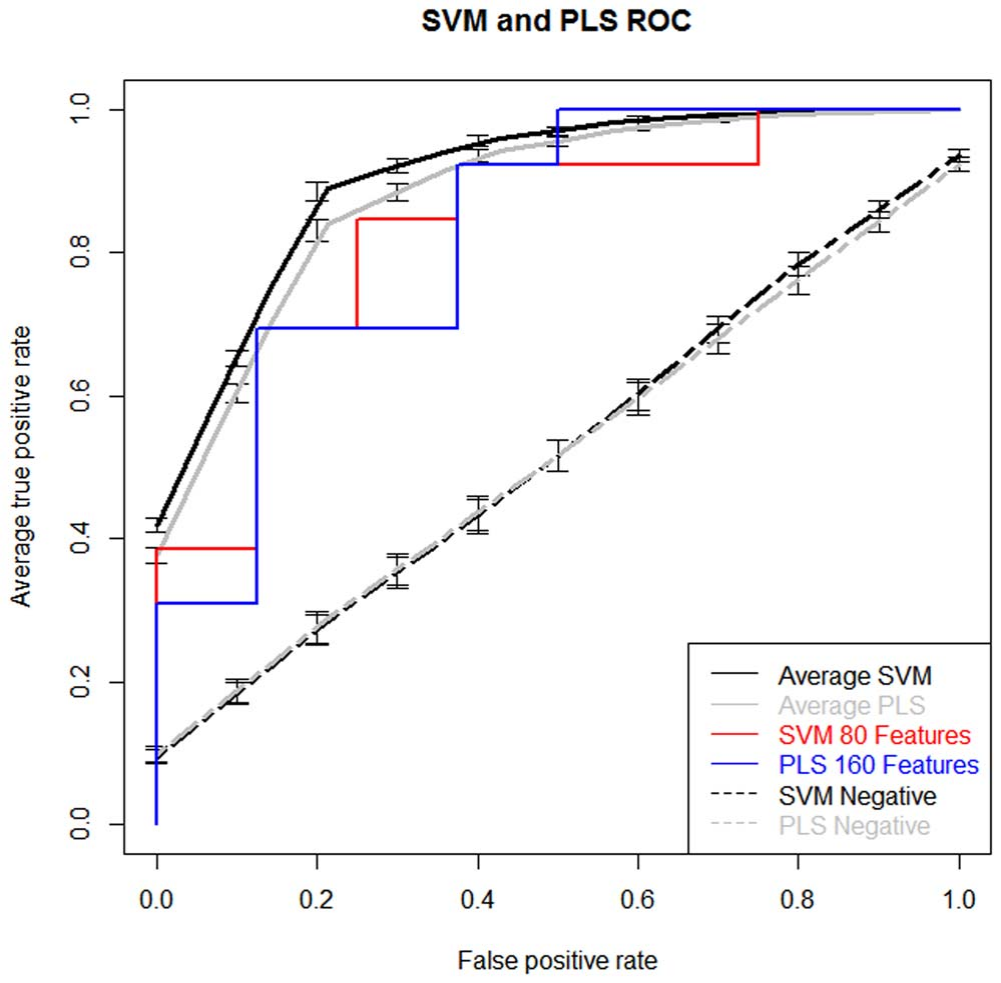
ROC curve performance of the classification models from the training and validation sets. The average of 100 iterations of the classifier for the best performing feature sets following recursive feature elimination comparing ASD vs. TD samples (Black and Grey Lines). The blue (PLS) and red (SVM) lines are ROC curves of the best performing validation feature subsets. Vertical bars represent the standard error of the mean.

Different subsets of features, created based on the weighted VIP scores, were evaluated independently of the outer cross-validation loop using the 21-sample independent validation set. The 80-feature SVM model described above had a classification prediction accuracy of 81%, a sensitivity of 85%, a specificity of 75% and an AUC of 84% (Figure 4, red line; Table 3). The best performing PLS model, comprised of 160 variables, had an accuracy of 81%, a sensitivity of 92%, a specificity of 63% and an AUC of 0.81% (Figure 4, blue line; Table 3). Detailed results are shown in Table S3. The results suggest that at least 40 features are needed to reach an accuracy of 70% and that a range of 80 to 160 features had the best performance with this independent validation sample set as well as the training set of samples.

**Table 3:** Classifier performance metrics based on predictions on the independent 21-sample validation set, showing the feature sets with the highest accuracy.

Model	Feature No.	Accuracy	Sensitivity	Specificity	AUC
SVM	80	0.81	0.85	0.75	0.84
PLS	160	0.81	0.92	0.63	0.81

Feature No. corresponds to the number of the ordered, ranked VIP features that were evaluated. Table S3 shows the results for all feature sets.

### Confirmation of Metabolite Chemical Structures

The chemical identities of the 7 LC-MS mass features that were confirmed by LC-HRMS-MS are shown in Table 4. Included in the metabolites confirmed by LC-HRMS-MS or targeted GC-MS was homocitrulline, which was decreased in ASD patients. Other metabolites showing significant up or down regulation in our study include: aspartate, glutamate, DHEAS, citric acid, succinic acid, methylhexa-, tetra- and hepta-decanoic acids, isoleucine, glutaric acid, 3-aminoisobutyric acid and creatinine. These are listed in Table 4 and represent a variety of molecular classes including amino acids, organic acids, sterols, and fatty acids.

**Table 4:** Confirmed metabolites.

Analytical Platform	Metabolite	Feature ID	HMDB ID [61]	Fold Change (ASD/TD)	p-value (ASD vs. TD)	FDR	SVM Rank	PLS Rank
HILICpos	homocitrulline	M190T512	HMDB00679	0.67	<0.001	0.059	1	1
C8neg	2-hydroxyvaleric acid	M117T127	HMDB01863	0.80	0.0289	0.53	33	26
HILICpos	cystine	M241T774	HMDB00192	0.91	0.0277	0.532	87	121
GCMS	aspartic acid	GCMS_aspartic.acid	HMDB00191	1.33	<0.001	0.086	34	14
HILICpos	isoleucine	M132T248	HMDB00172	0.76	0.0351	0.541	60	69
HILICpos	creatinine	M114T262	HMDB00562	0.88	0.0471	0.576	57	75
GCMS	serine	GCMS_serine	HMDB00187	1.16	0.00275	0.267	137	118
HILICneg	4-hydroxyphenyllactic acid	M181T66	HMDB00755	0.84	0.0344	0.541	47	11
GC-MS	citric acid	GCMS_citric.acid	HMDB00094	0.91	0.0492	0.580	84	16
GC-MS	glutamic acid	GCMS_glutamic.acid	HMDB00148	1.28	0.00144	0.188	15	47
GC-MS	lactic acid	GCMS_indol.3.lactate	HMDB00671	0.87	0.0181	0.457	55	52
C8neg	DHEA sulfate	M367T736	HMDB01032	2.55	0.00152	0.188	11	67
GC-MS	glutaric acid	GCMS_glutaric.acid	HMDB00661	1.36	0.00492	0.322	27	15
GC-MS	5-hydroxynorvaline	GCMS_X5. Hydroxy norvaline.NIST	HMDB31658	1.27	0.0457	0.576	177	163
GC-MS	heptadecanoic acid	GCMS_heptadecanoic.acid.NIST	HMDB02259	0.81	0.0270	0.527	135	110
GC-MS	5-aminovaleric acid lactam	GCMS_X5.aminovaleric.acid.lactame	HMDB11749	2.43	0.00211	0.22	127	62
GC-MS	succinic acid	GCMS_succinic.acid	HMDB00254	1.11	0.0457	0.576	175	164
GC-MS	myristic acid	GCMS_myristic.acid	HMDB00806	0.76	0.00892	0.371	24	27
GC-MS	2-hydroxyvaleric acid	GCMS_X2.hydroxyvaleric.acid	HMDB01863	1.41	0.0406	0.564	179	171
GC-MS	methylhexadecanoic acid	GCMS_methylhexadecanoic.acid	NA	0.82	0.0399	0.564	160	120
GC-MS	3-aminoisobutyric acid	GCMS_X3.aminoisobutyric.acid	HMDB02166	1.19	0.0473	0.576	176	176

Statistically significant metabolites from the 61-sample training set with chemical structures confirmed by LC-HRMS-MS or GC-MS.

## Discussion

Our metabolomic approach was not biased toward possible biochemical pathways other than by the separation and detection limits of the analytical methods used. We used robust VIP scores and recursive feature elimination to estimate that between 80 and 160 mass features are required to produce an optimal predictive signature in this set of patients. The predictive signatures in this study are the result of modeling a 62-patient training set, then applying those models to predict a 21-patient validation set. This approach has resulted in the discovery of a biochemically diverse set of metabolites that might be useful in distinguishing individuals at risk for ASD. It is difficult to determine how generalizable these predictive signatures will be in the broader population, given the small sample size. Larger studies need to be performed in order to assess and refine a signature into a clinical diagnostic. Several of the metabolites identified so far in these signatures point to biological mechanisms that have been previously identified as having a role in the etiology of ASD. Our signatures will most likely represent a portion of the metabolic changes that will be critical in the diagnosis of ASD through metabolic end points.

### Identification of metabolites previously associated with ASD

Examples of metabolites showing significant up or down regulation in the current study that have been previously associated with autism include:

- Tricarboxylic acid cycle associated molecules including citric acid (decreased) and succinic acid (increased) were found to be significantly altered in the ASD participants. Elevations in urinary succinate [16], [17] and decreased urinary citrate [37] in children with autism have been previously reported.
- Fatty acids have previously been observed to be decreased in the plasma of children with ASD, similar to our observations for methylhexa-, tetra- and hepta-decanoic acids [12]. Links between saturated fatty acid metabolism and oxidative stress have been reported in erythrocytes in children with ASD [38].
- 3-aminoisobutyric acid was increased in samples from participants with ASD. This is also consistent with previous findings [39].
- Creatinine was decreased in children with ASD and is consistent with the findings of Whitely *et al,* who observed similar changes in urinary creatinine in children diagnosed with PDD [40].

### Evidence for a role in mitochondrial dysfunction in ASD

The goal of this study was to evaluate biomarkers in blood. When metabolism is disrupted, active transporters and tissue specific differences in metabolism can cause different levels of the same metabolites in different biological compartments (plasma, urine, CSF, etc.). When discussing individual metabolites in the context of autism, it is important to recognize that autism is a systemic disease that affects other organ systems besides the brain. For example, serotonin levels have been reported to be elevated in blood in some patients with autism, and other evidence suggests that intracerebral serotonergic activity is decreased in ASD. Serotonin does not cross the blood brain barrier, and the brain has different enzymes for serotonin synthesis than peripheral tissues [41].

Many of the confirmed metabolites that are associated with ASD are relevant to aspects of mitochondrial biology. Mitochondrial disease or dysfunction may be a risk factor for autism [42]. In addition, several other observed metabolites are associated with other processes already proposed to be involved in ASD including oxidative stress [43] and energy production [44].

- Aspartate and glutamate levels in blood were significantly elevated in ASD, as has been observed in previous studies [45], [46]. Mutations in the aspartate/glutamate mitochondrial transporter, SLC25A12, have been previously associated with ASD [47]. This transporter is an important component of the malate/aspartate shuttle, a crucial system supporting oxidative phosphorylation, adenosine triphosphate production, and key metabolites for the urea cycle [47].
- DHEAS, the predominant plasma sterol, was increased in children with ASD. DHEA is known to affect mitochondrial energy production through inhibition of enzymes associated with the respiratory chain [48] with variable findings in children with ASD [49], [50].
- The branched chain amino acid isoleucine was reduced in samples from children with ASD versus TD children as observed by others [51]. Possible molecular mechanisms would include mutation in the branched chain amino acid kinase dehydrogenase (BCKD-kinase), a mitochondrial enzyme [52] as well as amino acids in energy metabolism [53].
- Glutaric acid levels were elevated in children with ASD. Increased urinary glutaric acid occurs in a variety of neuronal deficiencies such as glutaryl-CoA dehydrogenase (GCDH) deficiency. A significant portion of the glutaric acid metabolism takes place in the mitochondria [54].

### The potential relationship of the gut microbiome with ASD

This potential connection between the gut microbiome and ASD is also receiving considerable attention [55]. Metabolomic studies of urine from individuals with ASD have identified molecules associated with the microbiome such as dimethylamine, hippuric acid and phenylacetylglutamine [16], [17]. We observed decreased plasma levels of p-hydroxyphenyllactate, a metabolite associated with bifidobacteria and lactobacilli that is known to serve as an antioxidant both in the circulation and tissues [56]. We have yet to identify other microbiome related metabolites.

### Novel metabolic alterations in ASD

We identified novel statistically significant changes in some metabolites that had not been previously reported in other metabolomics profiling as well as identifying novel changes in metabolites that had never before been associated with ASD. Significant changes in the levels of aspartate, citrate, creatinine, DHEA-S, hydroxyphenyllactate, indoleacetate, isoleucine, glutamate and glutarate between ASD and TD individuals were identified in this study compared to previous metabolomics studies of urine metabolites where changes in these metabolites were not significant [17]. These differences could be related to transport and accumulation of metabolites in the urine compared to the blood, differences in the study populations, or application different LC-MS and GC-MS methodology allowing better detection of these metabolites in this study.

We also identified a new, previously undescribed potential ASD biomarker, homocitrulline. This metabolite was decreased in ASD patients and had the highest rank of all features in both SVM and PLS classification models. Homocitrulline is a poorly understood molecule which is known to be formed inside the mitochondria from lysine and carbamoyl phosphate. The decreased homocitrulline levels in the blood suggests that homocitrulline metabolism in the brain may also be disrupted, Homocitrulline levels are increased in urine and blood in patients with ornithine translocase (SLC25A15) deficiency which diverts carbamyl phosphate to react with lysine. These patients can exhibit behavioral abnormalities similar to ASD such as developmental delay, ataxia, spasticity, learning disabilities, cognitive deficits and/or unexplained seizures [57]. Rats treated with intracerebroventricular administration of homocitrulline are also observed to have disrupted brain redox status and energy metabolism [58], [59]. These observations suggest that elevated brain levels of homocitrulline are deleterious; however additional studies are needed to define the brain levels of homocitrulline and the potential role in the development of ASD.

## Summary

The current study profiled metabolites in blood plasma using metabolomics methods to evaluate the possibility that differences in the metabolite abundances might provide a metabolic signature that could prove useful in distinguishing individuals at high risk for developing ASD. The cohort of subjects enrolled in this study was carefully assembled to reflect a diagnosis of ASD by strict research criteria. Beyond careful clinical diagnosis, great pains were taken to ensure that fasting blood collection was obtained at the same time for all study participants and that complicating factors such as illness were minimized. We consider the current work as a proof of concept that there are predictive metabolic signatures which can be used to distinguish ASD and TD individuals.

Two independent statistical classification methods (PLS and SVM) were employed to determine the most influential metabolites and mass features that could be used to discriminate between ASD and TD individuals. Both classification modeling methods yielded relatively similar results with respect to maximum prediction accuracy of about 81% as evaluated by an independent 21-sample validation sample set. This was followed by recursive feature elimination to establish the minimal numbers of features needed for a predictive model. Interestingly, several of the key features for classification, such as homocitrulline, were common between the two methods indicating their importance in the development of future blood based diagnostics. It is clear that access to a larger sample set will be required to further validate and confirm the annotations of the key features. Metabolomics determines changes in small molecule metabolites that are reactants and products of endogenous biochemical processes as well as small molecules derived from diet, the gut microbiome and contact with the environment. Perturbations in their abundance can result not only from genomic and proteomic influences, but from environmental and epigenetic influences as well. A metabolomic approach may therefore provide enhanced predictive results by keying in on common, end stage metabolites rather than on specific genomic or proteomic determinants.

### Limitations

While the patient population was very well characterized, represented all severity levels of ASD and the blood samples were taken in a very systematic fashion, the total number of subjects (ASD = 52; TD = 30) was not large enough to definitively create a model for prediction of ASD or characterize metabolic differences that may be more highly associated with ASD subtypes.

Due to the small sample size, analysis of the data with respect to medication, sex, special diet, race, ethnicity or other potential confounding covariates was not conclusive. These are important considerations which require a larger sample size to address properly. A much larger study is currently in progress. Randomization methods were implemented to help prevent biasing the results based on any single covariate. The 4 misclassified patients in the 80 feature SVM model in the validation test set were Caucasian male and females whereas the Hispanic, Asian, and other ethnicity were predicted correctly.

Strong evidence of hemolysis, based on visual observation, was observed in 5 samples from ASD children. These samples were excluded from analysis Hemolysis can be a result of poor medical condition, but can also result from suboptimal blood collection and handling. However, Yin et. al. found that if EDTA tubes were placed on ice immediately, as was done in this protocol, the metabolome was very stable. Therefore, it remains possible, but unlikely, that minor hemolysis may confound the data by effecting some of the metabolite fold changes that were observed [60].

## Conclusions

This initial study provides proof of concept to further pursue development of metabolic biomarkers of ASD. We have demonstrated that a profile of altered metabolites in the blood plasma from a well-curated sample set from clinically diagnosed children with ASD and TD individuals between 4 and 6 years of age, can be detected by a combination of several MS-based metabolomic analyses. Statistical models developed from the derived metabolic data distinguished children with ASD from TD individuals with better than 80% accuracy in both the 61-sample training set and the 21 sample validation set.

The broad metabolite profiling methods developed here can also be employed to discover a wide variety of additional metabolites, leading to the determination of biochemical pathways and mechanisms that are involved in the etiologies of ASD, and advancing the understanding of autism in broader patient populations, eventually leading to new modes of therapy. Given the pronounced clinical and co-morbid features of ASD, it is possible that metabolic profiling of individual patients may enable individualized therapeutic approaches for improved outcomes.

### Future Endeavors

Further research is currently being carried out in much larger and younger patient populations to confirm these results, discover and confirm additional diagnostic metabolites and determine which are the most robust for evaluating ASD risk. We are also comparing metabolite profiles in clinically defined subtypes of ASD to determine whether predictive accuracy can be increased through better phenotyping of the ASD population. Analysis and consequential stratification will be performed based on covariables such as medication, sex, special diet, race, ethnicity, onset and co-morbid features such as gastrointestinal distress or seizure disorders may lead to a more accurate set of diagnostic metabolic profiles.

## Supporting Information

Table S1 **Metabolic features used in the classification models.** A table of all 179 model features.
(XLSX)

Table S2 **Results from the cross-validation (CV) training sets.**
(XLSX)

Table S3 **Classifier performance metrics based on predictions on the independent 21-sample validation set.**
(XLSX)

Table S4 **Patient gender and ethnicity.**
(XLSX)

Table S5 **Complete Feature Data.** All mass feature data used for modelling.
(XLSX)

## References

[1] American Psychiatric Association (2013) Desk Reference to the Diagnostic Criteria from DSM-5. 5th ed. Washington, D.C.: American Psychiatric Association.

[2] Centers for Disease Control and Prevention (2014) Prevalence of autism spectrum disorder among children aged 8 years - autism and developmental disabilities monitoring network, 11 sites, United States, 2010. MMWR Surveill Summ 63: 1–21 Available: http://www.ncbi.nlm.nih.gov/pubmed/24670961 24670961

[3] Dawson G, Rogers S, Munson J, Smith M, Winter J, et al (2010) Randomized, controlled trial of an intervention for toddlers with autism: the Early Start Denver Model. Pediatrics 125: e17–23 Available: http://www.ncbi.nlm.nih.gov/pubmed/19948568. Accessed 12 August 2013 19948568 10.1542/peds.2009-0958 PMC4951085

[4] Ganz ML (2007) The lifetime distribution of the incremental societal costs of autism. Arch Pediatr Adolesc Med 161: 343–349 Available: http://www.ncbi.nlm.nih.gov/pubmed/17404130 17404130 10.1001/archpedi.161.4.343

[5] State MW, Šestan N (2012) Neuroscience. The emerging biology of autism spectrum disorders. Science 337: 1301–1303 Available: http://www.ncbi.nlm.nih.gov/pubmed/22984058. Accessed 27 February 2013 22984058 10.1126/science.1224989 PMC3657753

[6] Berg JM, Geschwind DH (2012) Autism genetics: searching for specificity and convergence. Genome Biol 13: 247 Available: http://www.pubmedcentral.nih.gov/articlerender.fcgi?artid=3491377&tool=pmcentrez&rendertype=abstract 22849751 10.1186/gb-2012-13-7-247 PMC3491377

[7] Huguet G, Ey E, Bourgeron T (2013) The genetic landscapes of autism spectrum disorders. Annu Rev Genomics Hum Genet 14: 191–213 Available: http://www.ncbi.nlm.nih.gov/pubmed/23875794. Accessed 11 December 2013 23875794 10.1146/annurev-genom-091212-153431

[8] Bucan M, Abrahams BS, Wang K, Glessner JT, Herman EI, et al (2009) Genome-wide analyses of exonic copy number variants in a family-based study point to novel autism susceptibility genes. PLoS Genet 5: e1000536 Available: http://www.pubmedcentral.nih.gov/articlerender.fcgi?artid=2695001&tool=pmcentrez&rendertype=abstract. Accessed 7 August 2013 19557195 10.1371/journal.pgen.1000536 PMC2695001

[9] Wang K, Zhang H, Ma D, Bucan M, Glessner JT, et al (2009) Common genetic variants on 5p14.1 associate with autism spectrum disorders. Nature 459: 528–533 Available: http://www.pubmedcentral.nih.gov/articlerender.fcgi?artid=2943511&tool=pmcentrez&rendertype=abstract. Accessed 9 August 2013 19404256 10.1038/nature07999 PMC2943511

[10] Belgard TG, Jankovic I, Lowe JK, Geschwind DH (2014) Population structure confounds autism genetic classifier. Mol Psychiatry 19: 405–407 doi:10.1038/mp.2013.34 23546168 10.1038/mp.2013.34 PMC4123206

[11] Skafidas E, Testa R, Zantomio D, Chana G, Everall IP, et al. (2012) Predicting the diagnosis of autism spectrum disorder using gene pathway analysis. Mol Psychiatry in press. Available: http://www.ncbi.nlm.nih.gov/pubmed/22965006. Accessed 24 October 2012. 10.1038/mp.2012.126 PMC3966080 22965006

[12] El-Ansary AK, Bacha AG Ben, Al-Ayahdi LY (2011) Plasma fatty acids as diagnostic markers in autistic patients from Saudi Arabia. Lipids Health Dis 10: 62 Available: http://www.pubmedcentral.nih.gov/articlerender.fcgi?artid=3107800&tool=pmcentrez&rendertype=abstract. Accessed 6 August 2013 21510882 10.1186/1476-511X-10-62 PMC3107800

[13] James SJ, Melnyk S, Fuchs G, Reid T, Jernigan S, et al (2009) Efficacy of methylcobalamin and folinic acid treatment on glutathione redox status in children with autism 1–3. Am J Clin Nutr 89: 425–430. 19056591 10.3945/ajcn.2008.26615 PMC2647708

[14] Lee RWY, Tierney E (2011) Hypothesis: the role of sterols in autism spectrum disorder. Autism Res Treat 2011: 653570 Available: http://www.pubmedcentral.nih.gov/articlerender.fcgi?artid=3420784&tool=pmcentrez&rendertype=abstract. Accessed 23 August 2013. 22937253 10.1155/2011/653570 PMC3420784

[15] Damodaran LPM, Arumugam G (2011) Urinary oxidative stress markers in children with autism. Redox Rep 16: 216–222 Available: http://www.ncbi.nlm.nih.gov/pubmed/22005342. Accessed 23 August 2013 22005342 10.1179/1351000211Y.0000000012 PMC6837661

[16] Yap IKS, Angley M, Veselkov K a, Holmes E, Lindon JC, et al (2010) Urinary metabolic phenotyping differentiates children with autism from their unaffected siblings and age-matched controls. J Proteome Res 9: 2996–3004 Available: http://www.ncbi.nlm.nih.gov/pubmed/20337404 20337404 10.1021/pr901188e

[17] Ming X, Stein TP, Barnes V, Rhodes N, Guo L (2012) Metabolic perturbance in autism spectrum disorders: a metabolomics study. J Proteome Res 11: 5856–5862 Available: http://www.ncbi.nlm.nih.gov/pubmed/23106572 23106572 10.1021/pr300910n

[18] Dunn WB, Bailey NJC, Johnson HE (2005) Measuring the metabolome: current analytical technologies. Analyst 130: 606–625 Available: http://www.ncbi.nlm.nih.gov/pubmed/15852128. Accessed 19 August 2013 15852128 10.1039/b418288j

[19] Gross ML (1994) Accurate masses for structure confirmation. J Am Soc Mass Spectrom 5: 57 Available: http://link.springer.com/10.1016/1044-0305(94)85036-4 24222515 10.1016/1044-0305(94)85036-4

[20] Bruce SJ, Jonsson P, Antti H, Cloarec O, Trygg J, et al (2008) Evaluation of a protocol for metabolic profiling studies on human blood plasma by combined ultra-performance liquid chromatography/mass spectrometry: From extraction to data analysis. Anal Biochem 372: 237–249 Available: http://www.ncbi.nlm.nih.gov/pubmed/17964273. Accessed 12 August 2013 17964273 10.1016/j.ab.2007.09.037

[21] American Psychiatric Association (2000) Desk Reference to the Diagnostic Criteria from DSM IV. 4th ed. Washington, D.C.: American Psychiatric Association.

[22] Corbett BA, Kantor AB, Schulman H, Walker WL, Lit L, et al (2007) A proteomic study of serum from children with autism showing differential expression of apolipoproteins and complement proteins. Mol Psychiatry 12: 292–306 Available: http://www.ncbi.nlm.nih.gov/pubmed/17189958. Accessed 14 January 2013 17189958 10.1038/sj.mp.4001943

[23] Ashwood P, Corbett BA, Kantor A, Schulman H, Van de Water J, et al (2011) In search of cellular immunophenotypes in the blood of children with autism. PLoS One 6: e19299 Available: http://www.pubmedcentral.nih.gov/articlerender.fcgi?artid=3087757&tool=pmcentrez&rendertype=abstract. Accessed 21 January 2013. 21573236 10.1371/journal.pone.0019299 PMC3087757

[24] Jiye A, Trygg J, Gullberg J, Johansson AI, Jonsson P, et al (2005) Extraction and GC/MS analysis of the human blood plasma metabolome. Anal Chem 77: 8086–8094 Available: http://www.ncbi.nlm.nih.gov/pubmed/16351159 16351159 10.1021/ac051211v

[25] Fiehn O, Wohlgemuth G, Scholz M, Kind T, Lee DY, et al (2008) Quality control for plant metabolomics: reporting MSI-compliant studies. Plant J 53: 691–704 doi:10.1111/j.1365-313X.2007.03387.x 18269577 10.1111/j.1365-313X.2007.03387.x

[26] Smith CA, O'Maille G, Want EJ, Qin C, Trauger S a, et al (2005) METLIN: a metabolite mass spectral database. Ther Drug Monit 27: 747–751 Available: http://www.ncbi.nlm.nih.gov/pubmed/16404815 16404815 10.1097/01.ftd.0000179845.53213.39

[27] Horai H, Arita M, Kanaya S, Nihei Y, Ikeda T, et al (2010) MassBank: a public repository for sharing mass spectral data for life sciences. J Mass Spectrom 45: 703–714 Available: http://www.ncbi.nlm.nih.gov/pubmed/20623627. Accessed 23 August 2013 20623627 10.1002/jms.1777

[28] Orchard S, Montechi-Palazzi L, Deutsch EW, Binz P-A, Jones AR, et al (2007) Five years of progress in the Standardization of Proteomics Data 4th Annual Spring Workshop of the HUPO-Proteomics Standards Initiative April 23-25, 2007 Ecole Nationale Supérieure (ENS), Lyon, France. Proteomics 7: 3436–3440 Available: http://www.ncbi.nlm.nih.gov/pubmed/17907277. Accessed 1 May 2014 17907277 10.1002/pmic.200700658

[29] Smith CA, Want EJ, O'Maille G, Abagyan R, Siuzdak G (2006) XCMS: processing mass spectrometry data for metabolite profiling using nonlinear peak alignment, matching, and identification. Anal Chem 78: 779–787 Available: http://www.ncbi.nlm.nih.gov/pubmed/16448051 16448051 10.1021/ac051437y

[30] Prince JT, Marcotte EM (2006) Chromatographic alignment of ESI-LC-MS proteomics data sets by ordered bijective interpolated warping. Anal Chem 78: 6140–6152 Available: http://www.ncbi.nlm.nih.gov/pubmed/16944896 16944896 10.1021/ac0605344

[31] Haury A-C, Gestraud P, Vert J-P (2011) The influence of feature selection methods on accuracy, stability and interpretability of molecular signatures. PLoS One 6: e28210 Available: http://www.ncbi.nlm.nih.gov/pubmed/22205940. Accessed 1 May 2014 22205940 10.1371/journal.pone.0028210 PMC3244389

[32] Benjamini Y, Hochberg Y (1995) Controlling the false discovery rate: a practical and powerful approach to multiple testing. J R Stat Soc Ser B 57: 289–300.

[33] Wold H (1985) Partial least squares. In: Kotz S, Johnson NL, editors. Encyclopedia of statistical sciences. New York: Wiley, Vol. 6 . pp. 581–591.

[34] Cortes C, Vapnik V (1995) Support-vector networks. Mach Learn 20: 273–297 Available: http://link.springer.com/article/10.1007/BF00994018. Accessed 1 May 2014

[35] Kuhn M (2008) Building predictive models in R using the caret package. J Stat Softw 28: 1–26.27774042

[36] Sing T, Sander O, Beerenwinkel N, Lengauer T (2005) ROCR: visualizing classifier performance in R. Bioinformatics. 21: 3940–3941 Available: http://www.ncbi.nlm.nih.gov/pubmed/16096348. Accessed 29 April 2014 10.1093/bioinformatics/bti623 16096348

[37] Frye RE, Melnyk S, Macfabe DF (2013) Unique acyl-carnitine profiles are potential biomarkers for acquired mitochondrial disease in autism spectrum disorder. Transl Psychiatry 3: e220 Available: http://www.pubmedcentral.nih.gov/articlerender.fcgi?artid=3566723&tool=pmcentrez&rendertype=abstract. Accessed 9 January 2014 23340503 10.1038/tp.2012.143 PMC3566723

[38] Ghezzo A, Visconti P, Abruzzo PM, Bolotta A, Ferreri C, et al (2013) Oxidative Stress and Erythrocyte Membrane Alterations in Children with Autism: Correlation with Clinical Features. PLoS One 8: e66418 Available: http://www.pubmedcentral.nih.gov/articlerender.fcgi?artid=3686873&tool=pmcentrez&rendertype=abstract. Accessed 7 August 2013 23840462 10.1371/journal.pone.0066418 PMC3686873

[39] Adams JB, Audhya T, McDonough-Means S, Rubin RA, Quig D, et al (2011) Nutritional and metabolic status of children with autism vs. neurotypical children, and the association with autism severity. Nutr Metab (Lond) 8: 34 Available: http://www.pubmedcentral.nih.gov/articlerender.fcgi?artid=3135510&tool=pmcentrez&rendertype=abstract 21651783 10.1186/1743-7075-8-34 PMC3135510

[40] Whiteley P, Waring R, Williams L, Klovrza L, Nolan F, et al (2006) Spot urinary creatinine excretion in pervasive developmental disorders. Pediatr Int 48: 292–297 Available: http://www.ncbi.nlm.nih.gov/pubmed/16732798. Accessed 22 July 2013 16732798 10.1111/j.1442-200X.2006.02207.x

[41] Whitaker-Azmitia PM (2005) Behavioral and cellular consequences of increasing serotonergic activity during brain development: a role in autism? Int J Dev Neurosci 23: 75–83 Available: http://www.ncbi.nlm.nih.gov/pubmed/15730889. Accessed 12 November 2013 15730889 10.1016/j.ijdevneu.2004.07.022

[42] Marazziti D, Baroni S, Picchetti M, Landi P, Silvestri S, et al (2012) Psychiatric disorders and mitochondrial dysfunctions. Eur Rev Med Pharmacol Sci 16: 270–275 Available: http://www.ncbi.nlm.nih.gov/pubmed/22428481 22428481

[43] Rossignol DA, Frye RE (2012) A review of research trends in physiological abnormalities in autism spectrum disorders: immune dysregulation, inflammation, oxidative stress, mitochondrial dysfunction and environmental toxicant exposures. Mol Psychiatry 17: 389–401 Available: http://www.pubmedcentral.nih.gov/articlerender.fcgi?artid=3317062&tool=pmcentrez&rendertype=abstract. Accessed 5 February 2013. 22143005 10.1038/mp.2011.165 PMC3317062

[44] Blaylock RL (2009) A possible central mechanism in autism spectrum disorders, part 2: immunoexcitotoxicity. Altern Ther Health Med 15: 60–67 Available: http://www.ncbi.nlm.nih.gov/pubmed/19161050 19161050

[45] Shinohe A, Hashimoto K, Nakamura K, Tsujii M, Iwata Y, et al (2006) Increased serum levels of glutamate in adult patients with autism. Prog Neuropsychopharmacol Biol Psychiatry 30: 1472–1477 Available: http://www.ncbi.nlm.nih.gov/pubmed/16863675. Accessed 15 July 2013 16863675 10.1016/j.pnpbp.2006.06.013

[46] Moreno-Fuenmayor H, Borjas L, Arrieta A, Valera V, Socorro-Candanoza L (1996) Plasma excitatory amino acids in autism. Invest Clin 37: 113–128. 8718922

[47] Napolioni V, Persico AM, Porcelli V, Palmieri L (2011) The mitochondrial aspartate/glutamate carrier AGC1 and calcium homeostasis: physiological links and abnormalities in autism. Mol Neurobiol 44: 83–92. 21691713 10.1007/s12035-011-8192-2

[48] Safiulina D, Peet N, Seppet E, Zharkovsky A, Kaasik A (2006) Dehydroepiandrosterone inhibits complex I of the mitochondrial respiratory chain and is neurotoxic in vitro and in vivo at high concentrations. Toxicol Sci 93: 348–356 Available: http://www.ncbi.nlm.nih.gov/pubmed/16849397. Accessed 9 January 2014 16849397 10.1093/toxsci/kfl064

[49] Strous RD, Golubchik P, Maayan R, Mozes T, Tuati-Werner D, et al (2005) Lowered DHEA-S plasma levels in adult individuals with autistic disorder. Eur Neuropsychopharmacol 15: 305–309 Available: http://www.ncbi.nlm.nih.gov/pubmed/15820420. Accessed 23 August 2013 15820420 10.1016/j.euroneuro.2004.12.004

[50] Tordjman S, Anderson GM, McBride PA, Hertzig ME, Snow ME, et al (1995) Plasma androgens in autism. J Autism Dev Disord 25: 295–304 Available: http://www.ncbi.nlm.nih.gov/pubmed/7559294 7559294 10.1007/BF02179290

[51] Arnold GL, Hyman SL, Mooney RA, Kirby RS (2003) Plasma amino acids profiles in children with autism: potential risk of nutritional deficiencies. J Autism Dev Disord 33: 449–454 Available: http://www.ncbi.nlm.nih.gov/pubmed/12959424 12959424 10.1023/a:1025071014191

[52] Novarino G, El-Fishawy P, Kayserili H, Meguid N a, Scott EM, et al (2012) Mutations in BCKD-kinase lead to a potentially treatable form of autism with epilepsy. Science 338: 394–397 Available: http://www.ncbi.nlm.nih.gov/pubmed/22956686. Accessed 24 October 2012 22956686 10.1126/science.1224631 PMC3704165

[53] Valerio A, D'Antona G, Nisoli E (2011) Branched-chain amino acids, mitochondrial biogenesis, and healthspan: an evolutionary perspective. Aging (Albany NY) 3: 464–478 Available: http://www.pubmedcentral.nih.gov/articlerender.fcgi?artid=3156598&tool=pmcentrez&rendertype=abstract 21566257 10.18632/aging.100322 PMC3156598

[54] Müller E, Kölker S (2004) Reduction of lysine intake while avoiding malnutrition–major goals and major problems in dietary treatment of glutaryl-CoA dehydrogenase deficiency. J Inherit Metab Dis 27: 903–910 Available: http://www.ncbi.nlm.nih.gov/pubmed/15505398 15505398 10.1023/B:BOLI.0000045775.03183.48

[55] Mulle JG, Sharp WG, Cubells JF (2013) The gut microbiome: a new frontier in autism research. Curr Psychiatry Rep 15: 337 Available: http://www.ncbi.nlm.nih.gov/pubmed/23307560. Accessed 7 August 2013 23307560 10.1007/s11920-012-0337-0 PMC3564498

[56] Beloborodova N, Bairamov I, Olenin A, Shubina V, Teplova V, et al (2012) Effect of phenolic acids of microbial origin on production of reactive oxygen species in mitochondria and neutrophils. J Biomed Sci 19: 89 Available: http://www.pubmedcentral.nih.gov/articlerender.fcgi?artid=3503878&tool=pmcentrez&rendertype=abstract. Accessed 6 August 2013 23061754 10.1186/1423-0127-19-89 PMC3503878

[57] Palmieri F (2004) The mitochondrial transporter family (SLC25): physiological and pathological implications. Pflugers Arch 447: 689–709. 14598172 10.1007/s00424-003-1099-7

[58] Viegas CM, Busanello ENB, Tonin AM, de Moura AP, Grings M, et al (2011) Dual mechanism of brain damage induced in vivo by the major metabolites accumulating in hyperornithinemia-hyperammonemia-homocitrullinuria syndrome. Brain Res 1369: 235–244 Available: http://www.ncbi.nlm.nih.gov/pubmed/21059345. Accessed 26 August 2014 21059345 10.1016/j.brainres.2010.10.112

[59] Sokoro A a H, Lepage J, Antonishyn N, McDonald R, Rockman-Greenberg C, et al (2010) Diagnosis and high incidence of hyperornithinemia-hyperammonemia-homocitrullinemia (HHH) syndrome in northern Saskatchewan. J Inherit Metab Dis 33 Suppl 3: S275–81 Available: http://www.ncbi.nlm.nih.gov/pubmed/20574716. Accessed 26 August 2014 20574716 10.1007/s10545-010-9148-9

[60] Yin P, Peter A, Franken H, Zhao X, Neukamm SS, et al (2013) Preanalytical aspects and sample quality assessment in metabolomics studies of human blood. Clin Chem 59: 833–845 Available: http://www.ncbi.nlm.nih.gov/pubmed/23386698. Accessed 18 August 2014 23386698 10.1373/clinchem.2012.199257

[61] Kanehisa M (1997) A database for post-genome analysis. Trends Genet 13: 375–376. 9287494 10.1016/s0168-9525(97)01223-7

